# PTLD Burkitt Lymphoma in a Patient with Remote Lymphomatoid Granulomatosis

**DOI:** 10.1155/2012/239719

**Published:** 2012-01-19

**Authors:** A. Stravodimou, A. Cairoli, T. Rausch, R. Du Pasquier, P. Michel

**Affiliations:** ^1^Department of Oncology, University Hospital of Lausanne, Rue du Bugnon 21, 1011 Lausanne, Switzerland; ^2^Haematology Service, University Hospital of Lausanne, Rue du Bugnon 21, 1011 Lausanne, Switzerland; ^3^Institute of Pathology, University Hospital of Lausanne, Rue du Bugnon 21, 1011 Lausanne, Switzerland; ^4^Neurology Service, University Hospital of Lausanne, Rue du Bugnon 21, 1011 Lausanne, Switzerland

## Abstract

Posttransplant lymphoproliferative disorder (PTLD) is a potentially fatal complication of solid organ transplantation. The majority of PTLD is of B-cell origin, and 90% are associated with the Epstein-Barr virus (EBV). Lymphomatoid granulomatosis (LG) is a rare, EBV-associated systemic angiodestructive lymphoproliferative disorder, which has rarely been described in patients with renal transplantation. We report the case of a patient with renal transplantation for SLE, who presented, 9 months after renal transplantation, an EBV-associated LG limited to the intracranial structures that recovered completely after adjustment of her immunosuppressive treatment. Nine years later, she developed a second PTLD disorder with central nervous system initial manifestation. Workup revealed an EBV-positive PTLD Burkitt lymphoma, widely disseminated in most organs. In summary, the reported patient presented two lymphoproliferative disorders (LG and Burkitt's lymphoma), both with initial neurological manifestation, at 9 years interval. With careful reduction of the immunosuppression after the first manifestation and with the use of chemotherapy combined with radiotherapy after the second manifestation, our patient showed complete disappearance of neurologic symptoms and she is clinically well with good kidney function. No recurrence has been observed by radiological imaging until now.

## 1. Introduction

Posttransplant lymphoproliferative disorders (PTLDs) include a heterogenous group of lymphoid proliferation occurring in recipients of bone marrow and solid organ transplants associated with immunosuppressive drug administration. Transplant patients undergoing long-term and heavy immunosuppressive treatments display a higher risk of developing lymphomas than the general population. The incidence of PTLD is estimated to vary from 2% in renal transplant recipients to 5–9% in heart recipients [1]. Most are B cell in origin and associated with the Epstein-Barr virus (EBV). A manifestation in the CNS is documented in less than 10% of all EBV + PTLD cases. We report here the followup of a patient with lymphomatoid granulomatosis after renal transplantation [2] who developed PTLD Burkitt lymphoma 9 years later, both times presenting with neurological symptoms.

## 2. Case Report

The patient was diagnosed in 1977, at the age of 16, with systemic lupus erythematosus (SLE). In 1998 she underwent a renal transplantation for end-stage renal failure. The donor was Epstein-Barr virus (EBV) positive (IgG VCA IF positive, EBNA AC IF positive, IgM VCA IF negative), whereas the patient was EBV negative. Immediate posttransplant evolution was characterized by two acute rejection episodes, treated with methylprednisolone (500 mg i.v. twice daily), and a stenosis of the transplanted ureter, which needed surgical correction. The patient's therapy regimen included Cyclosporin A 200 mg p.o. twice daily, mycophenolate mofetil (MMF) 600 mg/m^2^ p.o. twice daily, and prednisone 30 mg p.o. daily. In the following months the patient developed an abducens nerve palsy, a peripheral facial nerve palsy with ageusia, a trigeminal hypesthesia, and a herpes zoster of T10-11 dermatomes, all on the left side. A cranial MRI in January 1999 showed a right frontal gadolinium enhancing lesion, and a lumbar puncture revealed lymphocytic pleocytosis (white blood cell count of 43/mm^3^ from which 90.5% lymphocytes, 0.5% plasmocytes, and 8.5% monocytes, CSF glucose count of 8 mmol/L, and total protein concentration of 1380 mg/L). The patient was still seronegative for EBV, but cerebrospinal fluid polymerase chain reaction (PCR) was positive for EBV. Meanwhile she complained for the first time of posterior chest pain. Chest X-ray was normal, but a CT scan showed a localized thickening of the pleura. The pleura biopsy showed multiple foci of severe inflammatory reaction with a mixed cell type, including lymphocytes, plasma cells, macrophages, and giant cells. Numerous blood vessels presented extensive vasculitis. Immunohistochemistry showed the inflammation to be made up of small T cells (CD3+) surrounded by foci of rare B cells (CD20+, IgG+) without any morphological criteria of malignancy. A rearrangement study for the T-cell receptor (TCR) gene (T cells), as well as search for clonal rearrangement of the IgH chain gene (B cells) by southern blot, failed to reveal any monoclonality. In situ hybridization for EBV (EBER 1/2) in the pleural biopsy material was negative. This histological picture of a polymorphous and granulomatous type of inflammatory reaction centered around vessels with nerve involvement, necrosis, and various numbers of large atypical cells was highly suggestive for the diagnosis of lymphomatoid granulomatosis [2]. Cyclosporin A and MMF were tapered by 50%  and prednisone increased up to 60 mg daily per os for about one month. MMF was then stopped and prednisone gradually decreased. The neurological symptoms resolved over several months. Eight years later the patient still has a functioning renal graft, on prednisone 5 mg per day and Cyclosporin A 100 mg twice daily p.o.. The patient seroconverted for EBV (IgG VCA positive by IF, EBNA AC IF negative, IgM VCA IF negative) more than a year after transplantation, once immunosuppression was minimal, without clinical symptoms.

In November 2008, the patient developed diplopia and left palpebral ptosis. A complete left III palsy with ptosis and mydriasis, a mild left peripheral facial nerve palsy with ageusia, and hypoesthesia in the first branch of the left trigeminal nerve were found. The corrected visual acuity was 0.8 in the left and 1.0 in the right eye. There were no meningeal signs. There was slight conjunctival injection but no exophtalmos. The patient had symmetrical hyperreflexia without other corticospinal signs.

**Figure 1:**
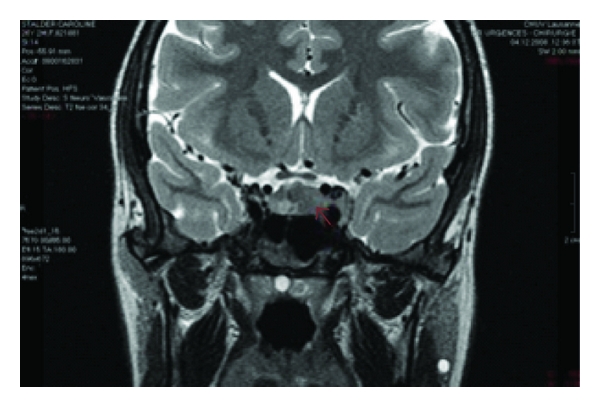
Cranial computed tomography shows a mass of 20 × 20 × 13 mm in the left part of the sella turcica.

**Figure 2:**
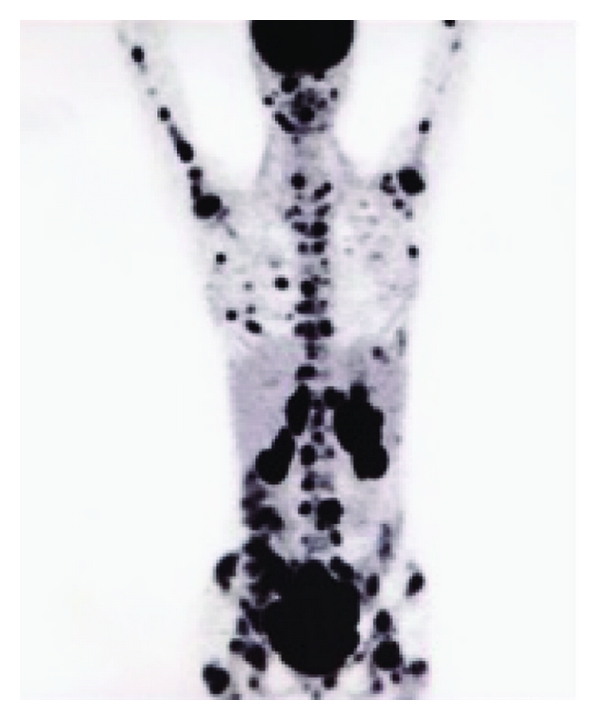
The positron emission tomography-computed tomography (PET-CT) shows a widespread pathological uptake of radiotracer (F-FDG), including bones, kidneys, and an intraperitoneal pelvic mass.

**Figure 3:**
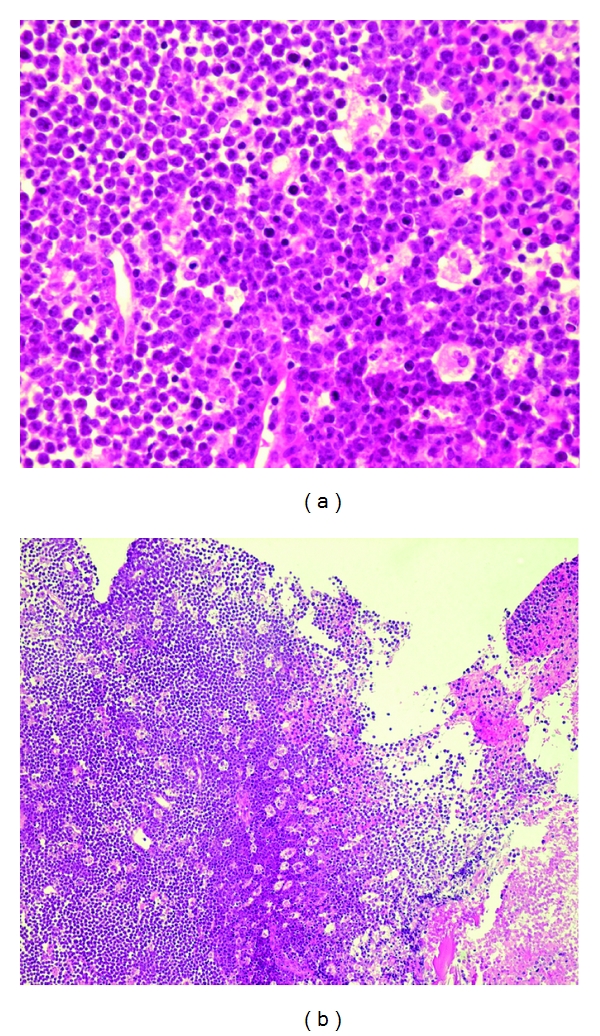
Tumor cells of large size admixed with numerous macrophages containing apoptotic bodies (“starry sky” aspect).

**Figure 4:**
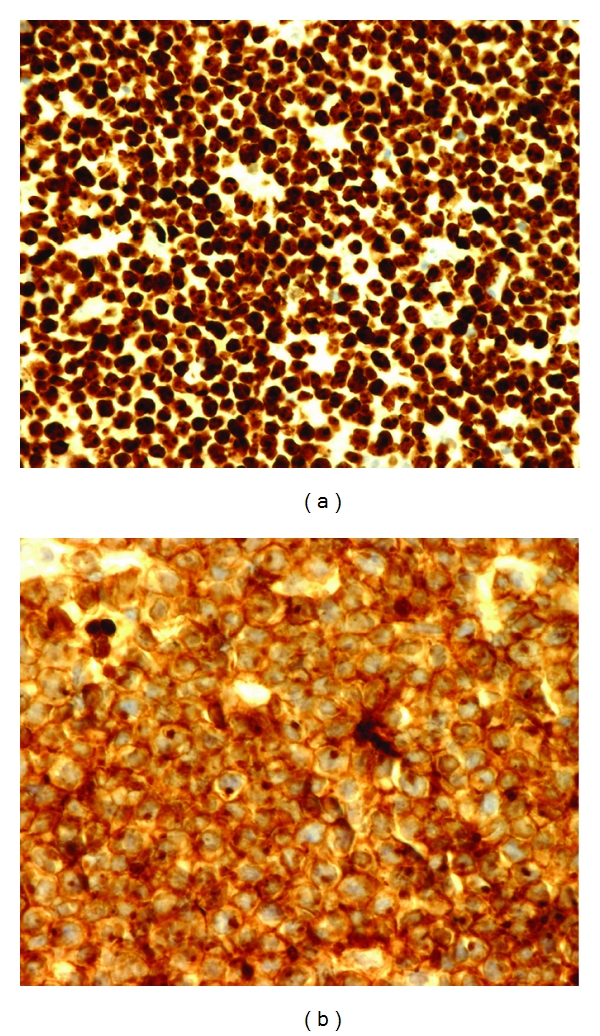
The proliferation rate (Ki67/MIB1) is 100% (a). The tumor cells expressed B lymphoid markers (not shown) with a coexpression of CD10 (b).

Cranial CT showed a contrast enhancing mass of 20 × 20 × 13 mm invading the left sella turcica (Figure 1), confirmed by an MRI. Complete blood count and peripheral smear were normal. Lumbar puncture revealed normal cell count (1 cell), mild increased protein concentration (393 mg/L), and normal glucose level (3.1 mmol/L). PCR studies performed on the peripheral blood and in the CSF were negative for cytomegalovirus (CMV), herpes virus simplex (HSV), and toxoplasmosis but demonstrated an EBV viral load of 155 900 copies/mL. Serum HIV test was negative. The hypophyseal hormones (LH, FSH, prolactin) were in the normal range. The positron emission tomography-computed tomography (PET-CT) showed a widespread disease (Figure 2). A transphenoidal biopsy of the mass showed a high-grade B Lymphoma of the Burkitt type (Figures 3 and 4) with a majority of medium-size cells with moderate cellular pleomorphism and variable nucleolar prominence. Mitosis and macrophages with apoptotic bodies were numerous. The immunohistochemical profile of these cells was CD20+ and CD79a+ with a coexpression of CD10 and Bcl6. The proliferation rate (Ki67) was 100%. A t(8; 14) translocation was detected by fluorescent in situ hybridization (FISH) analysis. The pelvic bone marrow aspiration showed no signs of medullary infiltration from the lymphoma. EBV was not searched in the tumor or in the bone marrow.

After diagnosis of PTLD Burkitt lymphoma, the immunosuppression was reduced and an intravenous chemotherapy associating Rituximab/CHOP (8 cycles), and an intermediate dose of Methotrexate (1.5 mg/m^2^), adapted to the renal function (5 cycles), was administrated. Vincristine had to be stopped because of new polyneuropathy, and cyclophosphamide was reduced by 25% after 5 cycles because of haematological toxicity. PET-CT after the end of chemotherapy was normalized except a small FDG-uptake in the ethmoid and sphenoid sinuses. Local radiation therapy was performed. The patient's neurological signs disappeared except for a mild residual facial palsy.

## 3. Discussion

Our patient with history of renal transplantation for SLE presented with two lymphoproliferative disorders, LG followed by Burkitt's lymphoma, both associated with EBV, at 9 years interval.

PTLD is one of the most important complications of organ transplantation, comprising 15% of all neoplasms [3], and long-term prognosis can be improved with early recognition and appropriate therapy. Despite numerous therapeutic advances, mortality rate reaches up to 60% in some reports. PTLDs usually have rapid onset, aggressive behavior, and are located extranodally. The exact relationship between lymphomatoid granulomatosis (LG) and posttransplant lymphoproliferative disorders (PTLDs) is not clear. Both are observed in immunodeficient patients and are Epstein-Barr virus driven [4]. Chronic EBV infection is probably the most important single event in the development of PTLD and LG, through activation of proto-oncogenes or surviving genes. EBV is associated with high proportion (89%) of posttransplant B-cell PTLD [5] and has been shown to promote B-cell proliferation by expressing an EBV-transforming protein (latent membrane protein 1) that engages the tumor necrosis factor receptor signal-transduction pathway to activate important regulators of cell growth, differentiation, or survival, such as nuclear factor kappa B and mitogen-activated protein kinases [4]. In particular, pretransplant EBV seronegativity followed by posttransplant seroconversion has been shown to be a major contributing risk factor for developing PTLD [6–8]. Monitoring the viral loads during the treatment may aid management because persistently elevated titers necessitate alternative treatment [9].

Although the association between EBV and PTLD is well established, the presence of EBV in tumor cells is not required for the diagnosis of PTLD. Negative findings in the biopsy material may reflect technical problems in retrieving the EBV genome from the rather rare infected B cells. This implies that, according to the international classification, any lymphoma arising in a posttransplant patient is considered to be a (variant of) PTLD [10]. Monitoring blood EBV load may detect early PTLD but cannot be used as a reliably early marker for PTLD. Assays are not standardized, and it is not known which tissue should be sampled or when to be tested. However, it is important to differentiate Burkitt-type PTLD from other types when deciding upon a course of treatment. Burkitt's lymphoma is not likely to be affected by a reduction in immunosuppression and characteristically requires adjuvant chemotherapy or radiation [11]. More effective treatment approaches to this disorder therefore need to be developed.
